# Matrons in Council: IX. Some Criticisms Examined. Needs of the Future

**Published:** 1917-10-06

**Authors:** 


### THE MATRONS' AND SISTERS' DEPARTMENT.

### MATRONS IN COUNCIL.

### IX. Some Criticisms Examined. Needs of the Future.

The suggestive and illuminating paper which appeared in The Hospital of August 4 contains a serious and somewhat severe indictment of the matrons of our training-schools for nurses.

The ideal of Florence Nightingale was a very high one; she considered that nursing should be in the hands of educated women, and the suggestions of the writer of "Sparks from a Nurse's Anvil" would seem to imply that the failure to live up to this ideal is in some part due to the attitude of matrons towards the education of their nurses, and to their lack of perception of the mental and professional needs of those whose training has been left in their hands. Naturally we ask ourselves if this is a just accusation, or at least how far, if at all, it is just, what are its causes, and what is its cure.

We hear often in the nursing profession that things are not what they were, that it appeals to a lower standard of education and of ideals, and that it has few attractions for really educated women. There is much truth in some of these criticisms, and, though matrons are not to be blamed for all, there seems to have been but little, if any, effort made by them to attract and encourage intelligent women to become nurses.

The enormously increased openings for women's work, the altered conditions of educational and professional standards, and the much larger number of women required as nurses, have all helped to increase the difficulty of keeping up the high level of attainment, but after all excuses and all changing circumstances have been allowed for, it is impossible to hold matrons free from blame in this matter. The "aloofness" of the average matron from her fellow-matrons has prevented discussion of methods, and has narrowed each training-school into its own small circle. There has been little or no co-ordination of thought or work; each has gone her own way, making her own improvements, small or great, with no further outlook than the teaching of the methods in vogue in her own hospital to her own pupils. Adaptability—a most valuable asset for every nurse—has been left out of mind altogether, because no realisation has been formed of the possible future requirements of each nurse who leaves that school. Probationers have been, to a great extent, looked upon as parts of a large machine, and so long as the whole worked smoothly and efficiently together, small attention has been given to the mental and ethical needs of each individual. From this has followed the lack of careful selection of those who show aptitude in special lines—in organisation, for instance, or administration or teaching—and this lack has often led to the loss of valuable work in these lines.

The reconstruction of matrons, therefore, will only result from a widened and enlarged educational system. Width and a large point of view will come from an extensive and comprehensive interchange of ideas, a knowledge of the special experience required for each variety of nursing needs, and a fixed intention to make the educational standard as high as it should be desired to keep those of practical work and of ideals.

The great needs of the future for the advancement of the nursing profession are:

1. *Education, Uniform and Systematised.*—At the present time the teaching of probationers is frequently, it may be said usually, done by some member of the staff whose sole experience of teaching has been picked up in the hospital. Every staff should be provided with one teacher who has either there or elsewhere been trained to teach, who is not worn and weary with ward work, housekeeping, or other duties, but who devotes her whole time and mind to the educational, as distinct from the practical, part of training. And so with pupils who are preparing for administrative duties; each school should have a teacher of domestic economy, housekeeping, and so on who gives her whole time to the pupils in her subject. In this way the special talent of each probationer could be developed and encouraged, and the deadening of unused faculties and the strain of continual, uncongenial drudgery would be removed.

2. *Regulation of Conditions of Work, and Secured Leisure for Study and Recreation.*—Fatigue is often given as the cause of the lack of interest in other matters, of which nurses as a class are accused; but such fatigue ought not to, and need not, exist. All continued work is apt to be fatiguing, but nursing, well ordered, need not wear out mind and body any more than any other continuous employment.

3. *Finance, Salaries, etc.*—This very serious and important subject can only be mentioned here, for it is impossible in so short a paper as this to deal with more than the fringe of this great problem.

Having pointed out where, in the writer's opinion, the present system has failed, in spite of much devoted and self-sacrificing work, it will be well, in the few words for which space can be found, to ask where we, as a profession, are to find the help for our needs. We see ourselves to-day great in numbers, filling important positions and doing important work, and absolutely without organisation, torn by dissensions, struggling for peace and progress which we have not yet found.

☛ Previous articles appeared in The Hospital of August 4, 11, 18, 25, and September 1, 15, 22, and 29.

In thinking over these points, it seems obvious that the advantages desired can only accrue to the profession when it learns to manage its own affairs. Trained nurses have come to the parting of the ways, and they must either bestir themselves and realise that they have to work out their own salvation, or see the profession most of us love, slowly deteriorate. They must cease to adopt the attitude of "I am of Apollos, and I of Cephas," and work heartily and with one mind for the good of the majority. It will no longer do to leave all the future in the hands of one or two leaders, however trusted those leaders may be. Those are not the real friends of nurses who assure them that their interests are safe in the hands of their matrons or of anyone but themselves or their elected representatives. Their real friends are those who clamour to them to waken from their apathy; to learn all they can, by reading and by discussion, of the opinions of their fellow-nurses; to consider both sides of all propositions, and to work heartily and with all their strength for what they decide to be right.

The proposed College of Nursing now offers the opportunity for self-government and for a wide and full education. It will provide what nurses desire for themselves, but it can only do this through the energy and the active help of the bulk of trained nurses. By these, and these alone, can the educational, physical, and ethical training of each individual nurse be secured.

There will be many thorny paths to travel, many pet theories to resign; we must all do—what is not easy to many women—sink the personal and consider only the principle. But, so united, the future will bring us to see things in a better proportion, and bring the profession, so nobly founded, to its deserved position.

